# The Treatment of Myocardial Degeneration

**Published:** 1905-11-25

**Authors:** 


### THE TREATMENT OF MYOCARDIAL DEGENERATION.

In the treatment of myocardial degeneration Dr. Arthur Foxwell[1] suggests it is necessary to remember that the change in the cardiac muscle and the atheroma of the largest arteries are accomplished facts which are hardly capable of remedy. The therapeutics of the condition are therefore restricted to attempts to modify the heart's output of strength by dilating or contracting the hypertrophied but healthy muscles of the brachial, radial, and smaller arteries. For the most part the patient's complaint is of symptoms which depend on inability of the heart to cope successfully with its difficulties. Hence the claim is to dilate the arterioles by the use of the trinitrin and potassium iodide, to rectify the metabolism by minute doses of calomel ($\frac{1}{30}$ to $\frac{1}{10}$ gr.) three to six times a day, and to improve nerve tone generally by full doses of strychnine. Digitalis and its allies are of doubtful value, and should only be given where there is rapidity or irregularity of the heart's action. At the same time, it must be remembered that if, as by the use of trinitrin and potassium iodide, the reduction of blood-pressure be excessive, the circulation may be rendered feeble and œdema may present itself. To hit the happy mean is difficult, and the desirable degree of blood-pressure varies with the individual patient and also in the same patient at different times.

Speaking on the same subject, Dr. Samuel West[2] remarks on the extraordinary susceptibility of degenerate hearts to the action of digitalis. In small doses this drug is of great service, but it has a curious cumulative effect upon degenerate cardiac muscle. This is seen in cases where the heart has been beating very rapidly (140 or so), when digitalis will promptly reduce the rate perhaps to 100 or less, and with great advantage to the patient. Then, without any appreciable change in the general condition, the pulse will suddenly fall to 40 or 50 beats per minute. In these circumstances further use of digitalis is certainly dangerous, and the administration of the drug should be promptly suspended.

[1] Brit. Med. Jour., October 21, 1905. [2] Ibid.

